# Supplementary material for: Use of Induced Pluripotent Stem Cells to Build Isogenic Systems and Investigate Type 1 Diabetes
Source: Front Endocrinol (Lausanne). 2021 Nov 9;12:737276. doi: 10.3389/fendo.2021.737276 (PMC8630743; doi:10.3389/fendo.2021.737276)
Supplement: Supplementary file 1 [file Image_1.pdf]

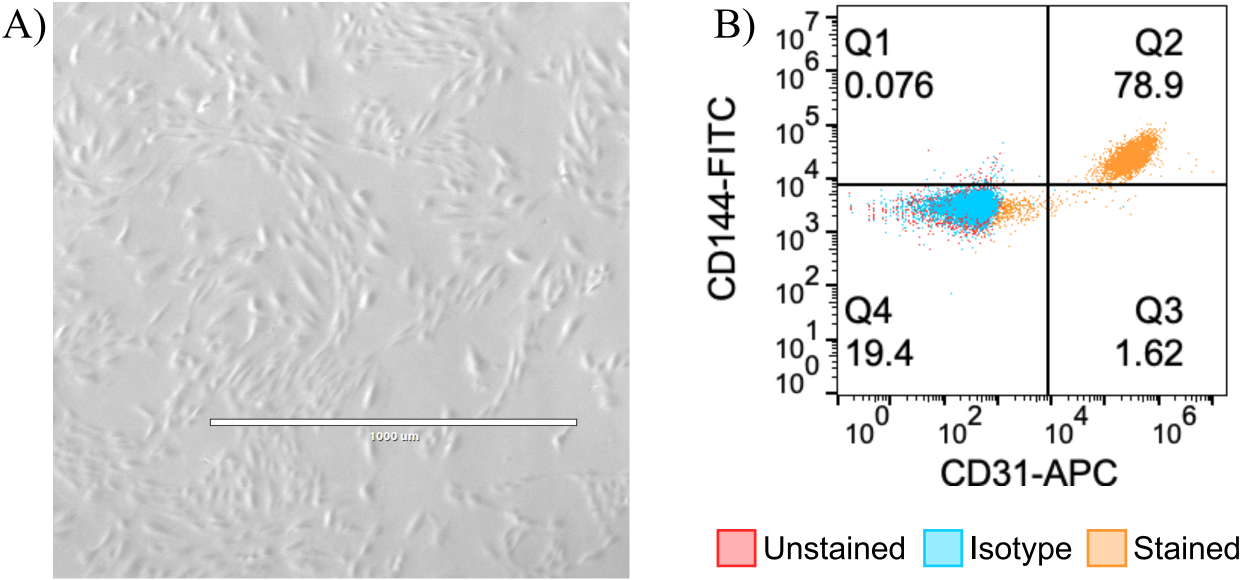

Supplementary Figure 1 | Morphology and marker expression of 2395 iPSC-EC. **(A)** 2395 iPSC-EC viewed under phase contrast microscopy display standard EC morphology. **(B)** Flow cytometry of 2395 iPSC-EC demonstrates that they express the EC markers, CD31 and CD144.
