# Supplementary material for: Use of Induced Pluripotent Stem Cells to Build Isogenic Systems and Investigate Type 1 Diabetes
Source: Front Endocrinol (Lausanne). 2021 Nov 9;12:737276. doi: 10.3389/fendo.2021.737276 (PMC8630743; doi:10.3389/fendo.2021.737276)
Supplement: Supplementary file 2 [file Table_1.docx]

**Supplemental Table 1. Antibody clones and catalog numbers.**

| Antigen | Fluorophore | Source | Clone | Assay | Company | Catalog # |
| --- | --- | --- | --- | --- | --- | --- |
| OCT4 | AF488 | mouse | 3A2A20 | FC | BioLegend | 653706 |
| SSEA-4 | PE | mouse | MC-813-70 | FC | BioLegend | 330406 |
| NANOG | AF647 | mouse | 16H3A48 | FC | BioLegend | 674210 |
| Nestin | PE | mouse | 25/NESTIN | FC | BD | 561230 |
| FoxA2 | PE | mouse | N17-280 | FC | BD | 561589 |
| Sox17 | AF488 | mouse | P7-969 | FC | BD | 562205 |
| NCAM | PE | mouse | HCD56 | FC | StemCell | 60021PE.1 |
| Brachyury | APC | goat | polyclonal | FC | R&D | IC2085A |
| NKX6.1 | PE | mouse | R11-560 | FC | BD | 563023 |
| PDX1 | AF488 | mouse | 658A5 | FC | BD | 562274 |
| Glucagon | BV421 | mouse | U16-850 | FC | BD | 565891 |
| C-peptide | AF647 | mouse | U8-424 | FC | BD | 565831 |
| PDX1 | n/a | goat | polyclonal | IF | R&D | AF2419 |
| anti-Goat | AF647 | donkey | polyclonal | IF | Thermo | A-21447 |
| C-peptide | n/a | rat | n/a | IF | DSHB | GN-ID4 |
| anti-Rat | AF488 | donkey | polyclonal | IF | Thermo | A-21208 |
| NKX6.1 | n/a | mouse | n/a | IF | DSHB | F55A10 |
| anti-mouse | AF555 | donkey | polyclonal | IF | Thermo | A-31570 |
| CD14 | PE | mouse | M5E2 | FC | BioLegend | 301806 |
| CD16 | FITC | mouse | eBioCB16 | FC | ThermoFisher | 11-0168-42 |
| CD64 | APC | mouse | 10.1 | FC | ThermoFisher | 17-0649-42 |
| CD11b | FITC | mouse | ICRF44 | FC | BioLegend | 301330 |
| CD68 | PE | mouse | eBioY1/82A | FC | ThermoFisher | 12-0689-41 |
| CD11c | APC | mouse | Bu15 | FC | BioLegend | 337208 |
| HLA-DR, DP, DQ | APC | mouse | Tü39 | FC | BioLegend | 361714 |

*FC = Flow Cytometry, IF = Immunofluorescence
